# Mycobacteria in Nail Salon Whirlpool Footbaths, California

**DOI:** 10.3201/eid1104.040936

**Published:** 2005-04

**Authors:** Duc J. Vugia, Yvonne Jang, Candi Zizek, Janet Ely, Kevin L. Winthrop, Edward Desmond

**Affiliations:** * California Department of Health Services, Berkeley, California, USA;; † California Department of Health Services, Richmond, California, USA;; ‡ Centers for Disease Control and Prevention, Atlanta, Georgia, USA

**Keywords:** Mycobacteria, *Mycobacterium fortuitum*, furunculosis, boils, footspa, whirlpool footbath, pedicure, nail salon, dispatch

## Abstract

In 2000, an outbreak of *Mycobacterium fortuitum* furunculosis affected customers using whirlpool footbaths at a nail salon. We swabbed 30 footbaths in 18 nail salons from 5 California counties and found mycobacteria in 29 (97%); *M. fortuitum* was the most common. Mycobacteria may pose an infectious risk for pedicure customers.

In October 2000, we investigated the first known outbreak of *Mycobacterium fortuitum* cutaneous infections acquired from whirlpool footbaths, also called footspas, at a nail salon in northern California (*1*). Over 100 pedicure customers had prolonged boils on the lower legs that left scars when healed (*1**,**2*). In the investigation, we swabbed the area behind the screen of the recirculation inlet in each of 10 footspas at the nail salon and recovered strains of *M. fortuitum* from all 10. Isolates from 3 footbaths and 14 patients were indistinguishable by pulsed-field gel electrophoresis and by multilocus enzyme electrophoresis (*1*).

Before this outbreak, *M. fortuitum* and other rapidly growing mycobacteria (RGM) caused localized cutaneous infections but usually in a healthcare-associated setting with surgical or clinical devices contaminated with water from the hospital or from the municipal water system (*3*). In the nail salon outbreak, we suspected that the mycobacteria entered the footspas through the municipal tap water and thrived in the large amount of organic debris accumulated behind the footspa recirculation screens. However, cultures of tap water at that nail salon later in the investigation yielded RGM in the *M. chelonae-abscessus* group but not *M. fortuitum* (*1*).

Since RGM are commonly found in municipal water systems (*4**–**6*), and since the nail care business is a $6 billion and growing industry in this country (*7*), we hypothesized that similar whirlpool footbath–associated RGM infections occurred sporadically but went unnoticed. Soon after we alerted the health communities to this outbreak, 3 cases of lower extremity RGM infections associated with 2 different nail salons were documented from southern California (*8*).

No study has been published on the prevalence of mycobacteria in whirlpool footbaths. To determine the prevalence of nontuberculous mycobacteria in this common nail salon equipment, we undertook a mycobacteriologic survey of footspas in nail salons in California from November to December 2000.

## The Study

Five large counties from different parts of California (Alameda, Sacramento, Orange, Riverside, and San Diego) participated in the survey. Counties chosen served large populations and had multiple nail salons with whirlpool footbaths. In each county, a team including the regional investigator of the California Bureau of Barbering and Cosmetology and a local public health professional visited selected nail salons. They assessed footspa equipment, cleaning solutions, and cleaning techniques and frequencies. Swab samples were also collected.

In each participating county, a convenience sample of ≥3 different nail salons equipped with whirlpool footbaths located in the town's main business section was randomly selected for the survey. Salon managers were questioned about cleaning and disinfection regimens of their footspas. Pedicure equipment time in service within the salon and make and model numbers of whirlpool pedicure equipment were noted. For each salon, 2 separate footspas were sampled, unless that salon only had 1 footspa, in which case only 1 swab was collected. Using a screwdriver, investigators removed the grate or filter screen covering the recirculation port in each footspa basin and inspected the area behind the screen for debris. A sterile, cotton-tipped culturette was used to swab this area and placed in standard transport medium.

At the California Microbial Disease Laboratory, each swab was removed from the transport medium, placed into a 50-mL tube containing 5 mL of sterile water, and the contents vortexed. The swab was then removed from the tube, and the remaining suspension was decontaminated with an equal volume of N-acetyl-L-cysteine-sodium hydroxide for 15 minutes, followed by neutralization with phosphate buffer and concentration by centrifugation (*9*). The sediment was spread onto Middlebrook 7H10 and Middlebrook 7H11/Mitchison 7H11 selective agar plates, Lowenstein-Jensen slants, Bactec 12B, and Bactec Mycobacteria Growth Indicator Tube 960 system (Becton-Dickinson, Sparks, MD, USA) liquid media.

The mycobacteria isolated were identified by ≥1 of the following methods: rapid DNA probes using nucleic acid hybridization (*10*), high performance liquid chromatography that produces mycolic acid patterns (*11*), and biochemical tests (*9*). *M. simiae* and *M. lentiflavum* were differentiated by urease activity and photochomogenicity; for both of these, *M. simiae* is positive and *M. lentiflavum* is negative (*12*). *M. smegmatis* group organisms were not differentiated to the species level. *M. mageritense* was identified by polymerase chain reaction restriction analysis at a Mayo Clinic laboratory and by DNA sequencing at the University of Texas Health Center in Tyler.

Thirty-one swabs were collected from 30 whirlpool footbaths in 18 nail salons from the 5 California counties. Twelve salons had ≥2 footspas; 6 had only 1 footspa. Of these 30 footspas, nontuberculous mycobacteria were cultured from 29 (97%). From 15 (50%), >1 mycobacterium species were isolated. No mycobacteria or other acid-fast organisms were isolated from 1 footspa that had only been in use for 11 days, whereas the positive footbaths had been in use for an average of 22 months (range 3–84 months).

Isolated from the whirlpool footbaths were 10 species of mycobacteria, 6 of which were RGM: *M. fortuitum*, *M. mucogenicum*, *M. smegmatis* group, *M. mageritense*, *M. neoaurum*-like RGM, and a pigmented unidentified nontuberculous mycobacterium (Table). *M. fortuitum* was the most frequently isolated mycobacterium, found in 14 (47%) of the 30 footspas surveyed and from all 5 counties. Rapid growers, including *M. fortuitum*, were found in 23 (76%) of the footspas. Slow-growing mycobacteria species were also recovered, including *M. avium* complex, *M. gordonae*, *M. simiae*, and *M. lentiflavum*. These species were less frequent than the rapid growers, except for *M. avium* complex, which was found in 5 (17%) of the footspas.

**Table:** Mycobacteria, by species, isolated from 30 whirlpool footbaths in 18 nail salons, California, 2000

Mycobacteria	N (%) of spas
Mycobacterium fortuitum*	14 (47)
M. mucogenicum*	7 (23)
M. mageritense*	6 (20)
*M. avium* complex	5 (17)
*M. smegmatis* group*	4 (13)
UNTM*	3 (10)
*M. simiae*	3 (10)
*M. gordonae*	2 (7)
*M. neoaurum*-like*	2 (7)
*M. lentiflavum*	2 (7)

*Rapid growers. UNTM, unidentified nontuberculosis mycobacteria.

Mycobacterial species vary in their ability to survive the selective NaOH decontamination step that was used in this study (*13*). Some solid media cultures that grew *M. fortuitum* had only a few colonies of this species; others had nearly confluent growth; and still other cultures grew in broth only, not solid media, making it impossible to determine the quantity of growth. For these reasons, quantitative information about the number of colonies present on solid media is not reported.

The whirlpool footbaths sampled came from 3 manufacturers. Disinfectants reportedly used included a variety of brand name products and chlorine bleach, used at intervals of 1 to 14 days. Five (17%) footspas reportedly did not go through any disinfectant process. Twenty-five (83%) of the surveyed footbaths had collected visible debris or slime behind the recirculation screen cover, either on the screen itself, on the tub surface, or both. Fifteen (50%) of footspa operators reported never having cleaned behind this screen. One footspa had no screen or visible debris; nevertheless, it tested positive for mycobacteria.

## Conclusions

Mycobacteria were isolated from virtually all pedicure spas surveyed, the sole exception being the footspa that had only been in service for 11 days. Mycobacteria were recovered whether or not disinfectants were reportedly used and whether or not debris was visible behind the recirculation screen.

RGM, *M. fortuitum* in particular, were the most frequently isolated mycobacteria. Our survey suggests that potentially pathogenic mycobacteria are widespread in these footspas across California. These organisms most likely were introduced into the footspas through the municipal water supply, where they colonized parts of the spas and probably the plumbing. Given that these whirlpool footbaths are widespread in California but similar infections known to date are rare, the presence of such mycobacteria alone may not be sufficient to cause pedicure customers to get cutaneous infections from using these spas. Our 2000 outbreak investigation noted an unusually large amount of debris behind the footspa recirculation screens, which might have provided a niche for mycobacteria to colonize and proliferate to large numbers. In that outbreak, customers who shaved their legs before using these implicated footspas were at higher risk for furunculosis than those who did not (*1*). However, some customers in that outbreak were infected even though they reportedly did not shave their legs before using the pedicure spas. Thus, while we documented the widespread presence of potentially pathogenic mycobacteria in footspas, the risk for infection remains unclear.

A limitation of this study is our inability to quantify the risk for cutaneous infection to pedicure customers despite finding widespread presence of RGM. We could not quantify reliably the amount of mycobacteria in each footspa with a positive culture. Furthermore, what we found in these footbaths may not be representative of other California counties or other states.

Nonetheless, our findings document the ubiquitous presence of potentially pathogenic mycobacteria among footspas of nail salons in California. The 2000 outbreak might have been a warning of what can happen again if this emerging infection is not adequately addressed. In 2004, a case report documented 2 cases of *M. mageritense* furunculosis associated with using footbaths at a nail salon in Georgia (*14*).

The California Board of Barbering and Cosmetology adopted new regulations in May 2001 requiring nail salons to follow specific cleaning and disinfection procedures to ensure that their footspa equipment is properly cleaned and maintained (*15*). Since our survey was conducted before these new regulations were implemented, further monitoring and research are needed to determine whether complying with the regulations will decrease the potential risk for mycobacterial cutaneous infections among pedicure customers.
